# Real-world effectiveness and safety of Sacituzumab Govitecan in metastatic triple-negative breast cancer: results from the multicenter retrospective observational SACISUR cohort in Southern Spain

**DOI:** 10.3389/fonc.2026.1717135

**Published:** 2026-02-24

**Authors:** Alejandro Falcón-González, Elisenda Llabrés-Valenti, Fernando Henao-Carrasco, Rocío Urbano-Cubero, Ana Godoy-Ortiz, Julio César Nieto-Ramírez, Ana Milena Vargas-Prado, Alba González-Haba, Braulio Martín-Calero, Cristina Morales-Estévez, María Valero-Arbizu, Natalia Chavarría-Piudo, Encarna González-Flores, Estefanía Casaut-Lora, Tamara Díaz-Redondo, Sara Estalella-Mendoza, Irene Zarcos-Pedrinaci, David Morales-Pancorbo, Ariadna Acosta-Sánchez, Elena Vicente-Rubio, Ana Gil-Torralvo, Mónica Cejuela-Solís, Rubén De Toro-Salas, Alicia Cano-Jiménez, Javier Pascual, Antonia Sánchez-Guisado, Josefina Cruz-Jurado, Icíar De la Fuente-Domínguez, Jose Andrés Rodríguez-García, Alberto Torres-Zurita, Javier Salvador-Bofill, Manuel Ruiz-Borrego

**Affiliations:** 1 Medical Oncoly Service, Hospital Universitario Virgen del Rocío, Sevilla, Spain; 2 Medical Oncoly Service, Hospital CHU Insular Materno Infantil, Gran Canaria, Spain; 3 Medical Oncoly Service, Hospital Universitario Virgen Macarena, Sevilla, Spain; 4 Medical Oncoly Service, Hospital Universitario de Jaén, Jaén, Spain; 5 Medical Oncology Unit, Hospital Universitario Virgen de la Victoria, Málaga, Spain; 6 Medical Oncoly Service, IBIMA Plataforma Bionand, Málaga, Spain; 7 Medical Oncoly Service, Biomedical Research Networking Center in Cancer (CIBERONC), Madrid, Spain; 8 Medical Oncoly Service, Hospital de Torrecárdenas, Almería, Spain; 9 Medical Oncoly Service, Hospital Doctor Negrín, Gran Canaria, Spain; 10 Medical Oncoly Service, Hospital Universitario de Badajoz, Badajoz, Spain; 11 Medical Oncoly Service, Centro Hospitalario de Canarias, Tenerife, Spain; 12 Hospital Universitario Reina Sofía, Instituto Maimónides de Investigación Biomédica, Córdoba, Spain; 13 Medical Oncoly Service, Hospital QuirónSalud-Oncoavanze, Sevilla, Spain; 14 Medical Oncology Service, Instituto de Investigacion E Innovacion Biomedica de Cadiz (INIBiCA), Institute for Biomedica Research and Innovation, Hospital Universitario de Jerez de La Frontera, Jerez, Cadiz, Spain; 15 Hospital Virgen de las Nieves, Instituto de Investigación biosanitaria ibs, Granada, Spain; 16 Medical Oncoly Service, Hospital de Puerto Real, Cádiz, Spain; 17 Medical Oncoly Service, Hospital Regional Universitario de Málaga, Málaga, Spain; 18 Medical Oncoly Service, Hospital Puerta del Mar, Cádiz, Spain; 19 Medical Oncoly Service, Hospital Costa del Sol de Marbella, Málaga, Spain; 20 Biomedical Research Institute of Málaga (IBIMA), Network for Research on Chronicity, Primary Care, and Health Promotion (RICAPPS), Instituto de Salud Carlos III, Madrid, Spain; 21 Medical Oncoly Service, Hospital Juan Ramón Jiménez, Huelva, Spain; 22 Medical Oncoly Service, Hospital Universitario Insular de Gran Canaria, Gran Canaria, Spain

**Keywords:** breast cancer, metastatic triple-negative, mTNBC, real-world data, Sacituzumab govitecan

## Abstract

**Background:**

Sacituzumab govitecan (SG) has demonstrated efficacy in metastatic triple-negative breast cancer (mTNBC) in clinical trials, but real-world data from routine clinical practice remains limited. This study aimed to evaluate the effectiveness and safety of SG in mTNBC patients in Southern Spain.

**Methods:**

This observational, multicenter, retrospective study included 159 mTNBC patients who received at least one cycle of SG between January 2022 and December 2023. Primary endpoints included real-world progression-free survival (rwPFS), overall survival (rwOS), and safety. Secondary endpoints explored treatment tolerability and management of adverse events. A pre-specified subset analysis focused on patients with central nervous system (CNS) metastases.

**Results:**

The median age of patients at diagnosis was 50 years (46.5% premenopausal). Median rwPFS was 4.6 months (95% CI 3.7-6.3) and rwOS was 10.9 months (95% CI 7.6-14.2). The objective response rate was 31.2%, with a disease control rate of 68.9%. Patients with CNS metastases (13.8%) had a median rwPFS of 2.3 months (95% CI 1.3-3.2). The most common adverse events were neutropenia (59.4%, grade 3-4: 30.4%) and diarrhea (49%, grade 3-4: 8.2%). Granulocyte colony-stimulating factor was administered as primary prophylaxis in 29.6% of patients and as secondary prophylaxis in 17.6%. Treatment discontinuation due to adverse events occurred in 5.7% of patients, while 43.4% required at least one dose reduction.

**Conclusion:**

SG demonstrated effectiveness and tolerability in mTNBC patients treated in routine practice, including those with CNS metastases, consistent with ASCENT trial results. These findings support the use of SG in clinical practice for mTNBC patients and suggest clinically meaningful activity and a manageable safety profile in patients with CNS involvement, despite the clinical challenges presented by this subgroup.

## Introduction

Triple-negative breast cancer (TNBC) - an aggressive molecular subtype defined by the absence of estrogen receptor (ER), progesterone receptor (PR), and human epidermal growth factor receptor 2 (HER2) expression – accounts for 15%-20% of invasive breast carcinomas (1, 2). This biologically heterogeneous malignancy exhibits a clinically aggressive phenotype with a poor prognosis (3), and median overall survival is limited to 12–18 months despite therapeutic advances (4). The absence of actionable targets, such as hormone receptors or HER2 overexpression, excludes the use of endocrine therapies and HER2-directed treatments, positioning cytotoxic chemotherapy as the predominant therapeutic approach for the majority of advanced cases.

Sacituzumab govitecan (SG) emerged as a therapeutic breakthrough following its April 2020 FDA accelerated approval for metastatic triple-negative breast cancer (mTNBC) refractory to ≥2 prior systemic therapies, including at least one for advanced disease. Full approval was obtained in 2021, and it is currently authorized in patients with pre-treated HR+/HER2- metastatic breast cancer. This antibody-drug conjugate (ADC) uses a humanized monoclonal antibody (hRS7 IgG1κ) to target trophoblast cell-surface antigen-2 (Trop-2) coupled to SN-38 – the active metabolite of irinotecan – though a hydrolysable CL2A linker. Upon Trop-2-mediated internalization, enzymatic cleavage releases SN-38, inducing topoisomerase I inhibition, DNA double-strand breaks, and subsequent apoptosis. SG demonstrated remarkable efficacy in this pretreated patient population in which most had received two to three prior lines of chemotherapy.

In the ASCENT trial (5), a total of 529 patients with triple-negative breast cancer were enrolled between November 2017 and September 2019 at 88 sites in 7 countries and were randomly assigned in a 1:1 ratio to receive SG or single-agent chemotherapy, with 61 patients with stable brain metastases (stable for at least 4 weeks before treatment) at baseline. Patients achieved median progression-free survival (PFS) of 4.8 vs 1.7 months (HR 0.41) and overall survival (OS) of 11.8 vs 6.9 months (HR 0.48) with SG or single-agent chemotherapy, respectively. The clinical impact of SG extends beyond TNBC, as evidenced by the TROPiCS-02 trial (6) in patients with hormone receptor-positive (HR+) and luminal HER2-negative locally recurrent inoperable or metastatic breast cancer, where it extended median overall survival to 14.4 months versus 11.2 months with standard chemotherapy (*p* = 0.020). Crucially, survival benefits occurred irrespective of Trop-2 expression levels, highlighting the broad efficacy of this therapeutic approach. These patients had received at least one previous endocrine therapy, a taxane, and a CDK4/6 inhibitor in any setting, along with 2 to 4 previous chemotherapy regimens for metastatic disease.

The imperative for real-world data in oncology arises from its unique capacity to validate therapeutic interventions across unselected patient populations and routine clinical practice – a critical consideration for mTNBC, where rapid disease evolution and therapeutic scarcity call for urgent insights beyond protocol-restricted trial cohorts. For this reason, the primary objective of this study was to characterize the clinical and demographic profile of patients with mTNBC receiving SG in a routine clinical practice setting in Southern Spain. Key endpoints included effectiveness, measured as real-world progression-free survival (rwPFS) and overall survival (rwOS), alongside an analysis of the safety profile, particularly the management of neutropenia and diarrhea. Treatment management patterns, including dose modifications and supportive care strategies, were also assessed. Special emphasis was placed on subpopulations, such as patients with brain metastases, given their exclusion from pivotal clinical trials and their distinct clinical challenges.

## Methods

### Study design and population

This was an observational, multicenter, retrospective cohort study conducted across healthcare institutions in Southern Spain as part of the SACISUR cohort study. We included patients with mTNBC who received at least one cycle of SG between January 1, 2022, and December 31, 2023. For inclusion, patients had to present TNBC according to standard American Society of Clinical Oncology–College of American Pathologists criteria (7) determined at the time of the most recent available biopsy. If a metastatic biopsy specimen was available, TNBC status was defined based on this sample; otherwise, the receptor status at initial diagnosis was used. Disease classification was based on the timing and extent of metastatic involvement at initial diagnosis. *De novo* stage IV disease was defined as patients presenting with metastatic disease (classified as stage IV according to the American Joint Committee on Cancer TNM staging criteria) within 3 months of their initial breast cancer diagnosis. *Relapse* after early stage was defined as patients initially diagnosed with non-metastatic breast cancer (stages I-III) with no evidence of distant metastases who had relapsed after surgery. Menopausal status was assessed based on clinical and laboratory parameters documented in the patient’s medical records. Premenopausal status was defined as women with regular menstrual cycles or amenorrhea for less than 12 months before receiving chemotherapy. Patients who had undergone bilateral oophorectomy were classified as postmenopausal regardless of age. Those with hysterectomy but preserved ovaries were classified based on age and hormonal parameters, if available.

Data collection included patient demographics, clinical characteristics, treatment patterns, adverse events, and survival outcomes.

### Data analysis

As this was an exploratory study with a descriptive aim of collecting, summarizing, and providing data on routine clinical management and outcomes in patients with mTNBC treated with SG, no prespecified hypothesis was made; therefore, sample size was not estimated.

Descriptive statistical analysis was performed on all study variables. For quantitative variables, measures of central tendency and dispersion were calculated (mean and standard deviation [SD]; median and interquartile ranges [IQR]). For qualitative variables frequencies and percentages were reported.

Data collected from medical records included: date of initial diagnosis, Eastern Cooperative Oncology Group (ECOG), histology, menopausal status, tumor grade, tumor stage, visceral involvement as well as CNS involvement, HR-negative or HR positive, HER2 intensity (0 vs low 1+ or 2+ with ISH negative), Ki-67 and BRCA status. Related to previous treatment we also included neo- or adjuvant therapy in early disease, previous treatments for advanced disease, and the associated response to each treatment.

Time-to-event endpoint analysis (rwPFS and rwOS) were estimated using the Kaplan-Meier method and compared between groups using the log rank test (Mantel-Cox), with supporting tests including Breslow (generalized Wilconxon) and Tarone-Ware tests for robustness verification. Median survival times with 95% confidence intervals (CI) were reported. rwPFS was defined as time from SG initiation until objective tumor progression or death or was censored at the last radiographic assessment in the case of patients without progression or death, while rwOS was calculated from treatment initiation to death from any cause. Patients without events were censored at the date of the last follow-up. The objective response rate (ORR) was assessed per investigator according to Response Evaluation Criteria in Solid Tumors (RECIST) version 1.1 (8), defined as the proportion of patients achieving either complete response (CR) or partial response (PR) as their best overall response, based on radiological assessments performed at regular intervals in accordance with institutional standards of care. The disease control rate (DCR) was defined as the proportion of patients achieving either an objective response (CR or PR) or stable disease lasting at least 6 months, as determined by radiological assessments performed according to institutional guidelines and evaluated using RECIST v1.1 criteria (8). A pre-specified subset analysis was performed on patients with central nervous system (CNS) metastases, a high-risk population typically underrepresented in clinical trials, to evaluate treatment outcomes in this specific subgroup of interest.

Missing data was not considered in the analyses. Statistical analyses were conducted using two statistical software packages: IBM SPSS Statistics version 31.0 and R version 4.x (R Foundation for Statistical Computing, Vienna, Austria). All R code was documented for reproducibility.

### Ethics approval

The study was conducted in accordance with the principles of the Declaration of Helsinki and approved by the Ethics Committee of Virgen Macarena University Hospital (Meeting 26/05/2023, ACTA 11/2023). All patient data were anonymized and processed according to applicable data protection regulations. All patients included in this retrospective observational study had previously provided general consent for the use of their clinical data in research studies as part of their standard care documentation.

## Results

### Patient characteristics

A total of 159 patients with mTNBC were included in this study. The median age at diagnosis was 50 years (range 21-77); 74 (46.5%) patients were premenopausal. The majority of patients (93.0%) had an ECOG performance status of 0–1 at treatment initiation. Regarding disease characteristics, 81.1% presented with initial localized stage I-III metastatic disease, while 17.6% had *de novo* stage IV. The median number of previous lines of therapy was 3 (range 2-8), with patients receiving a median of 7.9 cycles of SG. SG was administered as third-line or beyond in 86 (54.1%) patients, as second-line therapy in 66 (41.5%) patients, and as first-line therapy in 6 (3.8%) patients; 17% of patients had received immunotherapy as first-line treatment (**Table 1**).

**Table 1:** Clinical and demographic characteristics of the overall population and the subset of patients with CNS metastases.

Characteristic	Overall population N (%)	Cohort with CNS metastases N (%)
N	159	22
Age, median (IQR), y	50 (21-77)	45 (30-67)
ECOG		
0	61.6%	63.6%
1	31.4%	22.7%
2	5.7%	9.1%
3	1.3%	4.5%
Premenopausal	74 (46.5%)	11 (50.0%)
Ductal histology	134 (84.3%)	
HER2 status		
HER2 0	96 (60.4%)	11 (50.0%)
HER2 low	56 (35.2%)	11 (50.0%)
Visceral metastases at baseline	120 (75.5%)	21 (95.2%)
CNS disease	22 (13.8%)	22 (100.0%)
Disease stage at diagnosis, N (%)		
Recurrent disease	0	0
* De novo* or stage IV	28 (17.6%)	5 (22.7%)
Localized or stage I-III	129 (81.1%)	17 (77.3%)
Previous line of therapy, median (IQR)	3 (2-8)	2.4 (2-4)
SG third-line or beyond	86 (54.1%)	7 (31.8%)
SG second-line	66 (41.5%)	15 (68.2%)
SG first-line	6 (3.8%)	0 (0%)
SG cycles, median (IQR)	7.9 (1-52)	4.68 (1-27)
Neoadjuvant chemotherapy in early disease	97 (61.0%)	13 (59.1%)
pCR	17 (17.5%)^1^	12 (54.5%)^2^
Immunotherapy	7 (4.4%)	
Immunotherapy in first-line	27 (17.0%)	4 (18.2%)

^1^ Of the 97 patients who received neoadjuvant therapy.

^2^ Of the 13 patients who received neoadjuvant therapy.

CNS, central nervous system; ECOG, Eastern Cooperative Oncology Group Performance Status; IQR, interquartile range; N, number of subjects; pCR, pathological complete response; SG, sacituzumab govitecan.

Twenty-two study patients (13.8%) had CNS metastases at the time of SG initiation. The median age of this population was 45 years (range 30-67). This subgroup demonstrated a higher proportion of visceral disease involvement (95.2% vs 75.5% in the overall population) and a more extensive metastatic profile, with a higher proportion of *de novo* stage IV disease (22.7% vs 17.6% in the overall cohort). All had been treated with radiotherapy prior to SG (**Table 1**).

### Effectiveness

**Figure 1:**
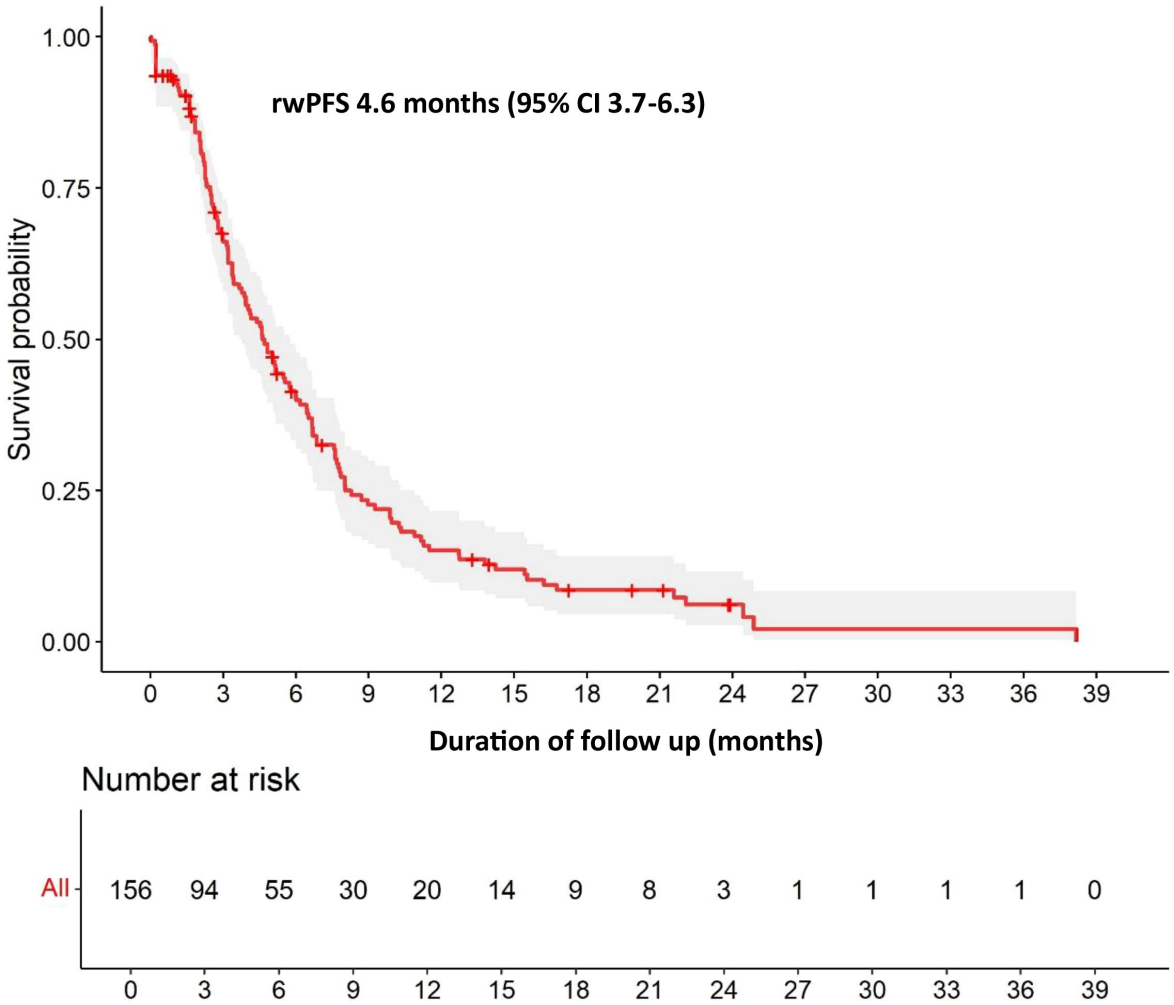
Overall rwPFS. rwPFS, real-world Progression-Free Survival.

**Figure 2:**
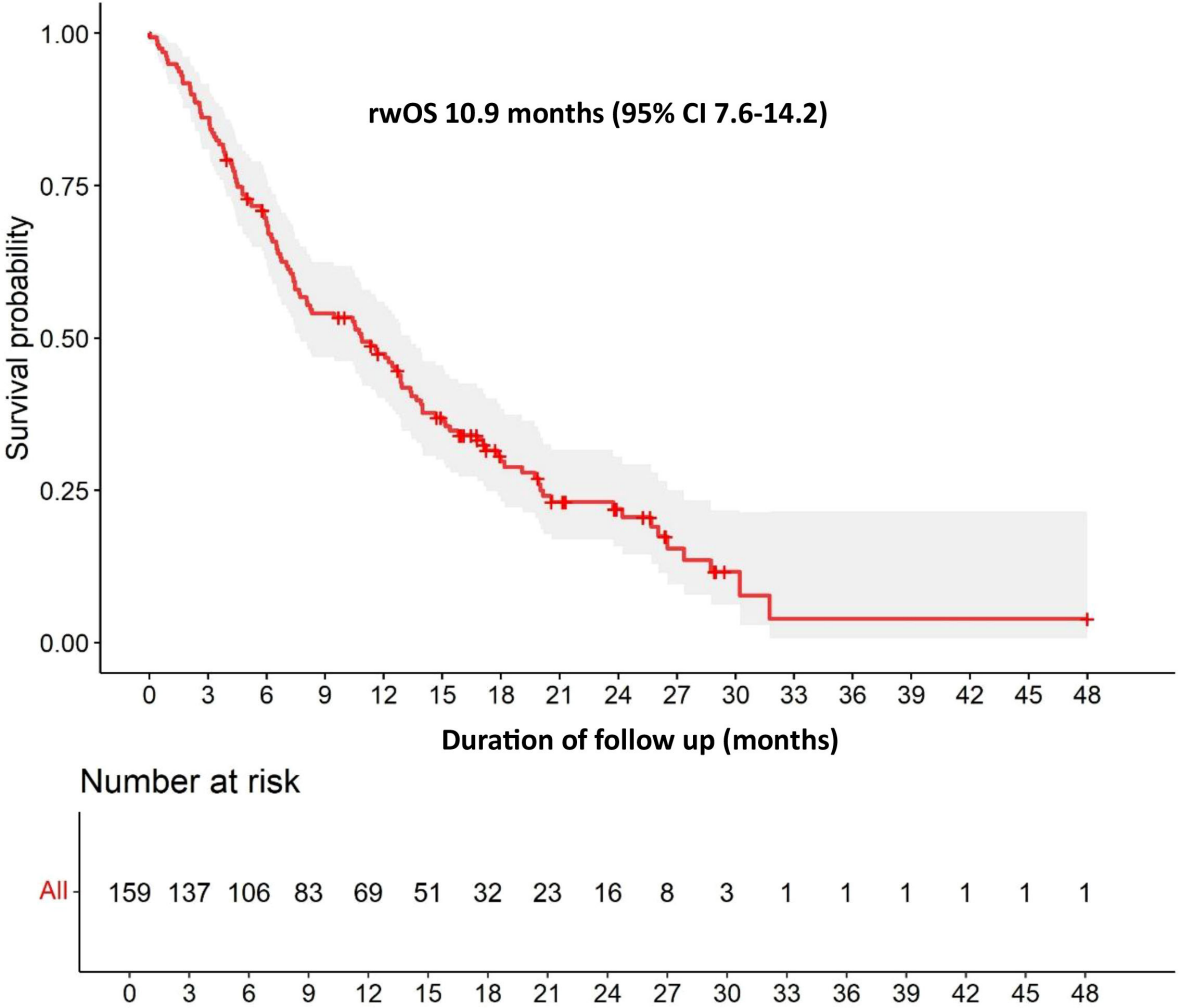
Overall rwOS. rwOS, real-world Overall Survival.

With a median follow-up of 11.6 months in the overall population, the median rwPFS was 4.6 months (95% CI 3.7-6.3) (**Figure 1**) and the median rwOS was 10.9 months (95% CI 7.6-14.2) (**Figure 2**). The ORR (CR or PR) was 31.2%, with a DCR (stable disease or objective response) of 68.9%.

**Figure 3:**
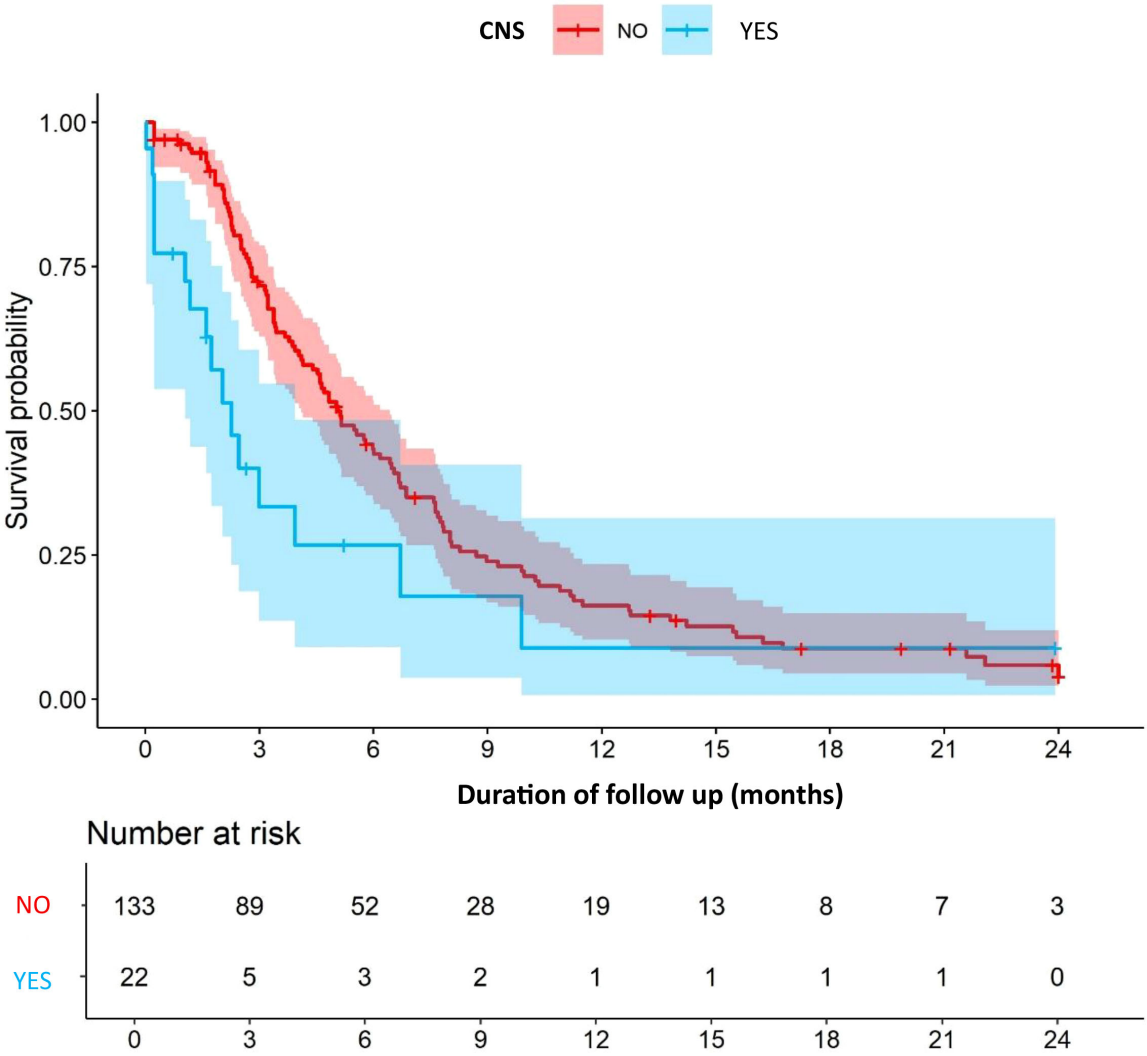
CNS metastases stratified rwPFS. CNS, central nervous system; rwPFS, Real-World Progression-Free Survival.

In patients with CNS metastases at baseline, the median follow-up was 6.0 months, during which the median rwPFS was 2.3 months (95% CI, 1.3–3.2) vs 5.1 months (95% CI, 4.1–5.9) in patients without CNS metastases (**Figure 3**). The ORR in this subgroup was 13.6%, with a DCR of 36.3%.

### Safety profile

SG was associated with diverse adverse events. Neutropenia was the most common, occurring in 59.4% of patients, with 30.4% experiencing grade 3–4 severity. Diarrhea affected 49.0% of patients, with 8.2% reporting grade 3–4 events. Nausea was observed in 45.3% of patients, although only 0.6% presented severe (grade 3-4) nausea. Elevation of liver enzymes (ALT/AST) was noted in 24.5% of patients, with 1.9% experiencing grade 3–4 elevations.

Granulocyte colony-stimulating factor (G-CSF) was administered as primary prophylaxis in 29.6% of patients and as secondary prophylaxis in 17.6%. Treatment discontinuation due to adverse events occurred in 5.7% of patients, while 43.4% required at least one dose reduction during their treatment.

In patients with CNS metastases, the most prevalent side effect of SG was also neutropenia, which occurred in 45.5% of patients, of which 27.2% experienced grade 3–4 severity. Diarrhea affected 36.8% of patients, with 9.1% reporting grade 3–4 events. Nausea was observed in 21.1% of patients, with 5.3% reporting grade 3–4 severity. Elevation of liver enzymes (ALT/AST) was noted in 30% of patients, although no grade 3–4 elevations were reported.

G-CSF was administered as primary prophylaxis in 18.2% of patients and as secondary prophylaxis in an additional 18.2%. Notably, no patients discontinued treatment due to adverse events, although 31.8% required at least one dose reduction during their treatment.

## Discussion

The results of our multicenter retrospective cohort have shown that SG is a safe and effective treatment option for patients with mTNBC in clinical practice. Our findings closely align with those of the pivotal ASCENT trial, even though our cohort included a broader patient population with potentially worse prognostic factors. The median rwPFS of 4.6 months and rwOS of 10.9 months observed in our study were comparable with the ASCENT trial results (median PFS 4.8 months, OS 11.8 months). Our ORR of 31.2% and DCR of 68.9% were in line with ASCENT’s ORR of 35% and DCR of 45%, suggesting preserved efficacy of SG in real-world settings. However, patients with CNS metastases demonstrated inferior survival outcomes, with a median rwPFS of 2.3 months compared with 5.1 months in patients without CNS involvement. This consistency with ASCENT is particularly noteworthy given our broader inclusion criteria, which encompassed patients with active brain metastases and poorer performance status. The slight differences in survival outcomes may be attributed to the inherent heterogeneity of real-world patient populations. For example, in our cohort 13.8% of patients presented CNS metastases, a subgroup that demonstrated inferior outcomes with a median rwPFS of 2.3 months. Other studies using real-world data have also reported similar results to those observed in clinical trials (9–12).

Our ORR and DCR compare favorably to those reported in ASCENT’, suggesting that the efficacy of SG is preserved, if not enhanced, in real-world clinical practice. The higher DCR in our study might reflect the potential for longer-term disease stabilization in a less strictly selected patient population. The consistency of these results across different studies based on routine clinical practice further supports the effectiveness of SG. For example, a retrospective analysis presented at the 42^nd^ Annual Miami Breast Cancer Conference (13) reported a median rwPFS of 5.0 months and rwOS of 11.3 months in patients receiving SG in the second-line or later setting, closely mirroring our findings and those of ASCENT (5).

A notably lower incidence of neutropenia was observed in our cohort compared with pivotal clinical trials. This reason for this is likely multifactorial: first, the use of primary prophylactic G-CSF was more common in our cohort, a strategy supported by recent data showing that prophylactic growth factor administration significantly reduces both the incidence and severity of SG-related neutropenia (14); second, recording neutropenia on day 8 of each cycle allowed clinicians to individualize each assessment and take timely action, such as delaying treatment or administering G-CSF to further reduce the risk and impact of neutropenic events (15).

This study has several strengths and limitations. Among the strengths, the multicenter, real-world design improves the generalizability of our findings across diverse healthcare settings in Southern Spain, providing valuable insights into the effectiveness and safety of SG in a broader patient population than typically included in clinical trials. The inclusion of patients with brain metastases, often excluded from pivotal trials, offers crucial data on this challenging subgroup. However, some limitations must also be mentioned, including the study’s retrospective design, which introduces potential biases in data collection and interpretation. The lack of stratification between stable and active brain metastases limits the granularity of the conclusions that can be drawn from this subpopulation. Additionally, the absence of a control group precludes direct comparisons with alternative treatments, and the relatively short follow-up period may not capture long-term outcomes or rare adverse events.

## Conclusion

In conclusion, this study demonstrates that SG shows clinically meaningful activity and is generally well-tolerated in patients with mTNBC treated in routine clinical practice. The efficacy outcomes, including median rwPFS of 4.6 months and rwOS of 10.9 months, closely mirror those observed in the pivotal ASCENT trial, despite the inclusion of patients with potentially worse prognostic factors such as active brain metastases and poorer performance status. The comparable ORR and higher DCR further support the preserved efficacy of SG in real-world settings. The study also highlights the challenges involved in treating patients with CNS metastases – a subgroup with inferior survival outcomes. However, the drug is considered both effective and safe in the treatment of this patient group.

## Data Availability

The original contributions presented in the study are included in the article/supplementary material. Further inquiries can be directed to the corresponding author.
